# The association of sleep patterns and depressive symptoms in medical students: a cross-sectional study

**DOI:** 10.1186/s13104-022-05975-8

**Published:** 2022-03-22

**Authors:** Katrin Dudo, Emilia Ehring, Stephan Fuchs, Sabine Herget, Stefan Watzke, Susanne Unverzagt, Thomas Frese

**Affiliations:** 1 Institute of General Practice & Family Medicine, Faculty of Medicine, Martin Luther University of Halle-Wittenberg, Magdeburger Straße 8, 06112 Halle (Saale), Germany; 2 Clinic for Psychiatry, Psychotherapy and Psychosomatics, University Hospital Halle (Saale), Julius-Kühn-Straße 7, 06112 Halle (Saale), Germany; 3 Department of General Practice, Medical Faculty of the University of Leipzig, Philipp-Rosenthal-Straße 55, 04103 Leipzig, Germany

**Keywords:** Medical student, Sleep, Depressive symptoms, Mental health

## Abstract

**Objective:**

Mental health is a fundamental aspect in ensuring the stable and successful professional life of future physicians. Depressive symptoms can negatively affect the work-life-balance and efficiency at work of medical students.

To date, there have been very few studies involving medical students that examine the association between single sleep characteristics and the outcome of the Beck Depression Inventory-II score. Therefore, the aim of the present study is to investigate this possible association. A classroom survey using socio-demographic characteristics, the Beck Depression Inventory-II, and the Pittsburgh Sleep Quality Index was conducted amongst students at a German medical school from December 2017 to September 2018. Data analysis was performed with descriptive statistics and binary logistic regression.

**Results:**

Of the students surveyed, 19% showed depressive symptoms with a Beck Depression Inventory-II score over 13 and 42% of these cases were moderate or severe. The occurrence of relevant depressive symptoms was associated with lower sleep quality, higher sleep latency, and the consumption of sleeping pills. In general, female students and students from abroad had a higher risk of depressive symptoms. Addressing these relevant findings in medical school can increase awareness of mental health.

## Introduction

Mental health is defined by the World Health Organization (WHO) as the “state of well-being in which an individual realizes his or her own abilities, can cope with the normal stresses of life, can work productively and is able to make a contribution to his or her community” [1]. It therefore forms a prerequisite for a stable and successful professional life in the medical field. Doctors often face emotionally stressful situations in their everyday work and carry a great responsibility for their patients. They are expected to act professionally, competently, and sensitively and to always be able to justify their actions. It is not uncommon for high levels of self-expectations and the demanding work environment to impact the mental health of physicians. [2]. Trainee doctors often face emotionally stressful situations early on in their medical degree programs. Numerous studies describe these challenges and the physical and mental health of medical students [3, 4]. In addition to anxiety disorders and behavioral disorders, depression is one manifestation of declining mental health [1]. In recent years, the large number of cases of depression among medical students has received more attention [5].

Sleep is crucial for the physical and mental regeneration of the body and therefore of great importance to mental health. A recent meta-analysis revealed that sleep quality is particularly poor amongst medical students, the highest prevalence of this phenomenon having been seen in students in Europe and America [6]. Poor sleep quality correlates to reduced academic performance [7] and is associated with the onset of anxiety, depression, and stress. A long sleep latency time (the time it takes an individual to fall asleep) may be an indicator of depression [8]. Also, reduced sleep duration has a negative effect on academic performance [9] and is more frequently associated with burnout [10].

To date, there have been very few studies involving medical students that examine the association between single sleep characteristics and the outcome of the Beck Depression Inventory-II (BDI-II). Therefore, the aim of the present study is to explore the possible association of sleep characteristics with depressive symptoms. The findings of this study will help to establish targeted interventions to improve the mental health of medical students.

## Main text

### Study design and data collection

A cross-sectional, comprehensive classroom-survey of medical students was conducted at a German medical university (Medical Faculty of the Martin-Luther-University Halle-Wittenberg, MLU in Saxony-Anhalt, Germany) between January 2018 and November 2018 to collect cross-sectional data across years one through five of the medical program. The survey was carried out anonymously. Students were given the option of completing the questionnaire during a compulsory session or taking it home to fill out and drop off in sealed containers located on campus. This study examined several different aspects of mental health in order to gain an overview of students’ mental health over the entire course of a medical degree program. In addition to the study, other projects additionally focused on aspects including the prevalence and risk factors of depressive symptoms or coping strategies [11, 12].

### Questionnaire

The questionnaire for the entire project was developed using a cross-disciplinary approach in collaboration with the University Hospital and Polyclinic for Psychiatry, Psychotherapy, and Psychosomatics of the University Hospital in Halle (Saale), MLU. The present study took into consideration various socio-demographic characteristics, BDI-II, and Pittsburgh Sleep Quality Index (PSQI).

The BDI-II [13] was used to identify depressive symptoms. It covers 21 items (mood, pessimism, sense of failure, loss of enjoyment, sense of guilt, sense of punishment, self-dislike, self-accusation, suicidal thoughts, crying, restlessness, loss of interest, indecisiveness, feelings of worthlessness, loss of energy, change in sleep habits, irritability, change in appetite, concentration difficulties, fatigability, loss of libido). Each item is placed onto a Likert scale with four possible answer choices varying in level of agreement with the statements. The answer to each statement is used to build a score: 0 to 13 points = no depressive symptoms, 14 to 19 points = mild depressive symptoms, 20 to 28 points = moderate depressive symptoms, > 28 points = severe depressive symptoms.

The PSQI was used to assess sleeping patterns. It contains 19 self-rated questions and 5 questions answered by a roommate on the case history. Eighteen of the nineteen self-rated questions (items) are used to calculate a score. They are divided into seven components (sleep quality, sleep latency, sleep duration, sleep efficiency, sleep disturbance, sleeping pill consumption, and daytime drowsiness) [14]. The individual components were considered in relation to the study population, as they are of greater importance in determining possible intervention strategies at the university than a differentiation between good and poor sleepers. The PSQI score was not considered in this context. The components were then assigned values as follows: poor sleep quality (consisting of poor and very poor sleep quality), sleep latency of more than 15 min, sleep duration of less than 7 h, sleep efficiency of less than 85%, sleep disturbances of more than 9 events per night, any use of sleep medication at all, and daytime drowsiness on more than 2 days per week.

### Statistical analysis

The SPSS 25.0 was used for data analysis. Descriptive statistics were used to describe the frequency, mean, and standard deviation of the collected data. A binary logistic regression was carried out with the BDI-II score as the dependent variable. The variable was dichotomized using the characteristics “no depressive symptoms” or “depressive symptoms”.

## Results

### Socio-biographical data

During the survey period, n = 1235 students were enrolled for the respective semesters. Of these students, 1124 attended their respective programs. The participation rate was n = 1103 (first semester n = 350; fourth semester n = 214; fifth semester n = 229; ninth semester n = 154; tenth semester n = 156) which corresponds to a response rate of 98.1%. Table 1 shows the distribution of gender and origin as a function of the BDI-II score. Students from abroad were more likely to suffer from at least mild symptoms of depression (BDI-II > 13) as well as female students when compared to their male classmates [15].

**Table 1:** Gender and origin as a function of the BDI-II score

	BDI-II ≤ 13; n (%)	BDI-II > 13; n (%)	Mean difference; % (95% CI)
Gender (n = 1103)			
Male	332 (85.8)	55 (14.2)	
Female	562 (78.5)	154 (21.5)	7.3 (2.5 to 11.7)
Origin (n = 1101)			
Germany	855 (81.9)	189 (18.1)	
Abroad	37 (64.9)	20 (35.1)	17 (5.6 to 30.2)

### Association between sleep and depressive symptoms

The worse the quality of sleep, the higher the risk for developing relevant depressive symptoms. While only 6.5% of medical students with “very good sleep quality” showed symptoms of depression, the risk for depressive symptoms increased by 7% in those students that indicated they had a “good quality of sleep”. A risk for relevant depressive symptoms as high as 76.9% was found amongst medical students with “very bad” quality of sleep. These observations were made for all 7 components of sleep. Table 2 provides a detailed list of all PSQI items as a function of the BDI-II score.

**Table 2:** PSQI items as a function of the BDI-II score

	BDI-II ≤ 13; n (%)	BDI-II > 13; n (%)	Mean difference; % (95% CI)
Sleep quality (n = 1096)			
Very good*	172 (93.5)	12 (6.5)	
Fairly good	610 (86.5)	95 (13.5)	7 (1.9–10.9)
Fairly poor	104 (53.6)	90 (46.4)	39.9 (31.7–47.4)
Very poor	3 (23.1)	10 (76.9)	70.4 (42.8–85.6)
Sleep latency in minutes (n = 1097)			
≤ 15*	530 (89.7)	61 (10.3)	
16–30	261 (78.4)	72 (21.6)	11.3 (6.4–16.5)
31–60	77 (60.2)	51 (39.8)	29.5 (21–38.4)
≥ 61	22 (48.9)	23 (51.1)	41.2 (26.8–55.3)
Sleep durationin hours (n = 1091)			
≥ 7*	688 (85.9)	113 (14.1)	
6	160 (72.4)	61 (27.6)	13.5 (7.4–20.1)
5	34 (57.6)	25 (42.4)	28.3 (16.3–41.2)
≤ 4	4 (40.0)	6 (60.0)	45.9 (17.1–69.2)
Sleep efficiency in % (n = 1091)			
≥ 85*	703 (85.0)	124 (15.0)	
75–84	141 (72.3)	54 (27.7)	12.7 (6.3–19.7)
65–74	34 (63.0)	20 (37.0)	22 (10.1–35.5)
≤ 64	8 (53.3)	7 (46.7)	31.7 (9.7–55)
Sleep disturbancesin events (n = 1072)			
None*	72 (92.3)	6 (7.7)	
1–9	761 (83.6)	149 (16.4)	8.7 (0.3–13.5)
10–18	40 (48.8)	42 (51.2)	43.5 (30.1–54.8)
19–27	–	2 (100)	92.3 (26–96.4)
Sleeping pill consumptionper week (n = 1094)			
None*	862 (83.3)	173 (16.7)	
< 1× per week	16 (51.6)	15 (48.4)	31.7 (15.1–48.6)
1–2× per week	8 (47.1)	9 (52.9)	36.2 (14.1–57.2)
≥ 3× per week	1 (9.1)	10 (90.9)	74.2 (45.5–82)
Daytime drowsinessin days (n = 1090)			
None*	218 (97.3)	6 (2.7)	
1–2	505 (86.9)	76 (13.1)	10.4 (6.5–13.7)
3–4	150 (62.0)	92 (38.0)	35.3 (28.7–41.7)
5–6	12 (27.9)	31 (72.1)	69.4 (54.3–80.7)

*Reference group

The binary logistic regression analysis shows in detail that sleep quality, consumption of sleeping pills, sleep latency, and gender are associated with depressive symptoms; poor sleep quality was associated with a four fold increased risk for the occurrence of depressive symptoms (OR = 3.99; 95% CI 2.67–5.96). Consumption of sleeping pills, longer sleep latency, and female gender were associated with a higher risk for depressive symptoms. The sleep duration showed little to no association with the outcome of the BDI-II score. Table 3 shows the regression analysis.

**Table 3:** binary logistic regression to explain the BDI-II score

	B	SE	Sig	Exp(B)	95% CI for Exp(B)	
					Lower	Upper
Sex	0.481	0.192	0.012	1.617	1.109	2.358
Sleep quality	1.384	0.204	< 0.001	3.991	2.674	5.959
Consumption of sleeping pills	1.166	0.318	< 0.001	3.209	1.722	5.980
Sleep latency	0.015	0.004	< 0.001	1.016	1.008	1.023
Sleep duration	− 0.149	0.079	0.060	0.862	0.738	1.006

## Discussion

Individual components of sleep are associated with depressive symptoms amongst medical students. The perception of sleep quality as “poor”, the consumption of sleeping pills, as well as prolonged sleep latency all negatively affect the mental health of medical students.

### PSQI

While there are multiple studies that divide participants into “good” and “poor” sleepers using their PSQI scores and assess medical students’ sleep quality in this way, there have been no relevant studies to date that pick out individual characteristics in detail and identify an association between them and the development of depressive symptoms. For each tested PSQI component, an increase in depressive symptoms was seen in those categories that reflected negative effects (see Table 2). This association has not been highlighted in other literature to date.

### Depressive symptoms among medical students

A meta-analysis carried out in 2016 showed that depression amongst medical students is a global issue [5]. The relationship between sleep quality and depressive symptoms among medical students has already been a focus of past literature [16–18]. Sleeping problems in general may be a predictor of depression, as the authors of a study among medical students in Bahrain found: students reported somatic symptoms rather than psychological problems [19]. In the present study, poor sleep quality and consumption of sleeping pills, as well as prolonged sleep latency and the female gender had the greatest association with the occurrence of depressive symptoms [20].

### Sleep quality

A meta-analysis on sleep quality amongst medical students showed that poor sleep quality affected 65% of European students [6]. In contrast, only one in five local students reported poor sleep quality. Although a standardized questionnaire was used, the results in the literature are generally quite different. These variations could be attributed to the curriculum or differences in the evaluation of the PSQI (score value versus subjective sleep quality) [21, 22].

### Consumption of sleeping pills

An assessment of the association between the consumption of sleeping pills and depressive symptoms among medical students cannot be found in the literature to date, as most studies only showed the frequency distribution of sleeping pill consumption. However, this is not always representative. For example, few MLU medical students (5.3%) reported taking sleeping pills (cf. 8.6% [23], 9% [24], 17% [25]), but an association with depressive symptoms was observed nonetheless.

### Sleep latency

Prolonged sleep latency as a contributing factor for depressive symptoms among medical students has hardly been studied. When the frequency distribution of sleep latency was investigated in studies, the mean values were similar to those in the present study, but not all studies were conducted solely with a cohort of medical students, making them less suitable for comparisons [22, 24, 26].

### Sleep duration

The optimal sleep duration for the young adult age group is 7 to 9 h, according to recommendations from the National Sleep Foundation [27]. While the average sleep duration among local medical students was 7 h, a sleep duration of less than 7 h was often reported in other studies [18, 21, 28].

## Conclusion

Individual sleep characteristics may be associated with depressive symptoms in medical students. Therefore, raising awareness about factors benefiting a healthy sleep is one possible way in which to support medical students. Addressing these topics in medical school can increase awareness of mental health.

## Limitations

The survey was performed at only one medical faculty in Germany. Although the curriculum is very standardized among German medical faculties and students within each faculty come from different regions of Germany, the monocentric design is a limitation.

Students were questioned at different points in time and a mere snapshot in time was evaluated. Further studies might consider conducing a longitudinal investigation.

The present study only investigates the association between sleep characteristics and depressive symptoms. Other sub-studies in this project highlight further aspects [11, 12].

Furthermore, poor sleep quality may not only lead to depressive symptoms but may itself be the result of depression. This causal relation is not investigated by this study.

## Data Availability

The datasets used and/or analyzed during the current study are available from the corresponding author upon reasonable request.

## Abbreviations

BDI-II: Beck-Depression Inventory-II
MLU: Martin Luther-University of Halle-Wittenberg
PSQI: Pittsburgh Sleep Quality Index
WHO: World Health Organization
